# Neuroprotective Effects of Myrtle Berry By-Product Extracts on 6-OHDA-Induced Cytotoxicity in PC12 Cells

**DOI:** 10.3390/antiox14010088

**Published:** 2025-01-13

**Authors:** Debora Dessì, Giacomo Fais, Paolo Follesa, Giorgia Sarais

**Affiliations:** 1 Department of Biomedical Science, University of Sassari, 07100 Sassari, Italy; d.dessi14@phd.uniss.it; 2 Interdepartmental Center of Environmental Science and Engineering (CINSA), University of Cagliari, Via San Giorgio 12, 09124 Cagliari, Italy; giacomo.fais@unica.it; 3 Department of Mechanical, Chemical and Materials Engineering, University of Cagliari, Piazza d’Armi, 09123 Cagliari, Italy; 4 Department of Life and Environmental Sciences, University of Cagliari, 09042 Cagliari, Italy

**Keywords:** neurodegenerative diseases, oxidative stress, myrtle berries, antioxidants, neuroprotection, Parkinson’s disease

## Abstract

The rising global focus on healthy lifestyles and environmental sustainability has prompted interest in repurposing plant-based by-products for health benefits. With increasing life expectancy, the incidence of neurodegenerative diseases—characterized by complex, multifactorial mechanisms such as abnormal protein aggregation, mitochondrial dysfunction, oxidative stress, and inflammation—continues to grow. Medicinal plants, with their diverse bioactive compounds, offer promising therapeutic avenues for such conditions. *Myrtus communis* L., a Mediterranean plant primarily used in liquor production, generates significant waste rich in antioxidant and anti-inflammatory properties. This study explores the neuroprotective potential of Myrtus berry by-products in a cellular model of neurodegeneration. Using PC12 cells exposed to 6-hydroxydopamine (6-OHDA), we assessed cell viability via MTT assay and measured reactive oxygen species (ROS) production using DCFDA fluorescence. Additionally, we analyzed the expression of genes linked to oxidative stress and neuronal function, including AChE, PON2, Grin1, Gabrd, and c-fos, by RT-PCR. Our findings reveal that Myrtus extract significantly protects against 6-OHDA-induced cytotoxicity, reduces ROS levels, and modulates the expression of key stress-related genes, underscoring its potential as a neuroprotective agent. These results highlight the therapeutic promise of Myrtus extracts in mitigating neurodegenerative processes, paving the way for future interventions.

## 1. Introduction

Parkinson’s disease (PD) is the second most prevalent neurodegenerative disorder after Alzheimer’s disease, characterized by the irreversible loss of dopaminergic (DA) neurons in the substantia nigra pars compacta [1]. This loss leads to a significant reduction of dopamine in the striatum, a region crucial for motor control, resulting in hallmark motor symptoms such as bradykinesia, rigidity, and resting tremor [2]. Non-motor symptoms, including cognitive decline, mood disturbances, and autonomic dysfunctions, often precede motor symptoms and highlight the systemic and multifaceted nature of the disease [3]. Oxidative stress and chronic neuroinflammation are central to PD pathogenesis [4,5]. A major source of oxidative stress is the overproduction of reactive oxygen species (ROS), primarily generated during mitochondrial oxidative phosphorylation [6]. Under normal conditions, ROS function as important signaling molecules; however, in PD, their excessive accumulation overwhelms antioxidant defenses, disrupts redox homeostasis, and damages lipids, proteins, and DNA [7]. The activation of oxidative stress also triggers inflammatory signaling, mitochondrial damage, and lysosomal dysfunction, collectively creating a vicious cycle of cellular degeneration. Recent studies indicate that targeting multiple nodes in these interconnected pathways could offer synergistic therapeutic benefits [8]. Indeed, the progression of neurodegenerative diseases like PD is strongly accelerated by the interplay between oxidative stress and neuroinflammation. Experimental evidence demonstrates that 6-hydroxydopamine (6-OHDA), a hydroxylated analog of dopamine, induces severe oxidative stress and DA neuronal damage in vitro and in vivo, making it a widely used model for PD pathogenesis [3,4,6]. By entering dopaminergic neurons through the dopamine transporter (DAT), 6-OHDA undergoes auto-oxidation, generating excessive ROS and reactive quinones. These products disrupt mitochondrial function, reduce ATP production, and activate apoptotic and necrotic pathways, ultimately causing neuronal death [9]. In vivo, unilateral injection of 6-OHDA into the medial forebrain bundle, striatum, or substantia nigra leads to region-specific DA neuronal loss, enabling the study of PD-related motor asymmetry [10]. In vitro, 6-OHDA-based models provide a controlled setting to investigate molecular mechanisms and screen potential neuroprotective agents [11]. This oxidative imbalance impairs mitochondrial Complex I, further decreasing ATP production and exacerbating ROS generation. Over time, these processes compromise cellular integrity, disrupt DNA repair, and provoke both apoptotic and necrotic pathways [12]. Markers of oxidative stress, such as malondialdehyde (MDA) and 4-hydroxynonenal (4-HNE), illustrate the extensive lipid peroxidation and mitochondrial dysfunction in PD [13]. Mitochondria are particularly vulnerable to oxidative damage, with early PD events including Complex I impairment, which limits ATP production and further increases ROS generation. This feedback loop of oxidative stress drives apoptotic pathways, directly contributing to dopaminergic neuron loss [14]. Peroxisomes also play a crucial role in maintaining cellular redox balance by detoxifying hydrogen peroxide (H_2_O_2_) through catalase activity. However, peroxisomal dysfunction, often accompanying mitochondrial impairment, intensifies oxidative stress and neurodegeneration, underlining the systemic nature of oxidative damage in PD [5]. Another hallmark of PD is the accumulation of α-synuclein, forming toxic aggregates known as Lewy bodies. These aggregates disrupt mitochondrial function and respiration by interacting with proteins like TOMM20 [15]. Oxidative modifications further stabilize α-synuclein aggregates, increasing mitochondrial and lysosomal stress. Lysosomal dysfunction then worsens this cascade by impairing the autophagic clearance of α-synuclein. Mutations in lysosomal enzymes, such as glucocerebrosidase (GBA1), exacerbate neuronal damage [13]. PD is also associated with impaired mitophagy, often linked to dysfunctions in the PINK1/parkin pathway, causing the buildup of damaged mitochondria, excessive ROS production, and elevated neuroinflammation [16,17]. Furthermore, oxidative stress compromises the blood–brain barrier (BBB), increasing its permeability to immune cells [18]. This infiltration amplifies neuroinflammation by releasing cytokines like TNF-α and IL-6, thereby escalating oxidative injury [5]. Dopaminergic neurons are particularly susceptible due to the auto-oxidation of dopamine, which generates quinones and hydrogen peroxide, further intensifying oxidative stress and α-synuclein pathology [17]. Because oxidative stress and neuroinflammation are pivotal in PD pathogenesis, plant-derived polyphenols have gained attention as potential therapeutic agents due to their antioxidant, anti-inflammatory, and neuroprotective properties [8,19,20]. Polyphenols, abundant in fruits, vegetables, and other plant-based foods, scavenge ROS, reduce lipid peroxidation, and modulate inflammatory pathways by inhibiting pro-inflammatory cytokines, such as TNF-α and IL-6 [5]. They also activate the Nrf2 pathway, boosting antioxidant enzyme expression, while supporting mitochondrial dynamics to preserve neuronal function. Emerging findings show that polyphenols can regulate autophagy and mitophagy, both essential for cellular homeostasis. By facilitating the removal of damaged organelles and proteins, polyphenols diminish cellular stress and inflammation, particularly in neurodegenerative disorders like PD [15]. Epidemiological data further suggest that raising antioxidant intake can slow disease progression and improve quality of life [21,22,23,24,25,26]. Additionally, polyphenols enhance brain function by promoting neurotrophic factors, activating survival pathways, and supporting cognition [27,28]. They may also improve cerebral blood flow and lower blood pressure, factors often impaired in neurodegeneration [29]. Synergistic effects between different types of polyphenols (e.g., flavonoids and phenolic acids) can amplify their neuroprotective actions, including PI3K/Akt pathway activation, which fosters neuronal survival and mitochondrial stability [8,17]. Among various sources of bioactive polyphenols, *Myrtus communis* L., a flowering plant native to the Mediterranean region, stands out for its rich content of flavonoids, phenolic acids, and tannins, all contributing to notable antioxidant and anti-inflammatory effects [30,31,32,33,34,35,36,37,38,39,40]. In recent years, food by-products have emerged as viable polyphenol sources in alignment with global sustainability goals, such as those outlined in the UN 2030 Agenda for Sustainable Development. Revalorizing these by-products addresses the dual challenge of reducing food waste while promoting health via nutraceuticals and functional foods [41,42,43]. Numerous studies have focused on developing green and efficient strategies for extracting bioactive compounds from plant wastes [44], transforming them into high-value derivatives, such as ingredients and functional foods rich in fiber, protein, and antioxidants. The bioactive potential of these extracts is well-documented, with properties including antioxidant, anti-inflammatory [45], anti-glycaemic [46], cardioprotective [47], antibacterial [48], and anticancer effects [49]. These effects are closely tied to the presence of diverse polyphenolic compounds that act in synergy to boost their biological activities. In this context, *Myrtus communis* L. stands out as a notable example, known for its broad pharmacological and therapeutic profile, including antioxidant [50], anti-inflammatory [51], antibacterial [52,53], gastroprotective [54,55], and hepatoprotective [56] properties. During the hydroalcoholic extraction to produce sweet liqueurs, substantial quantities of myrtle berry residues are generated, still retaining high levels of flavonoids, tannins, and phenolic acids [8]. In the Mediterranean area, especially Sardinia, about 200,000 tons of these myrtle by-products are produced annually [57]. Though often discarded, they represent a sustainable source of bioactive compounds with notable therapeutic potential [57]. Although the antioxidant and anti-inflammatory properties of fresh *Myrtus communis* L. extracts are well-documented, the potential of by-product extracts in neurodegenerative contexts, such as Parkinson’s disease (PD), remains largely unexplored [32]. This study is the first to assess the neuroprotective potential of *Myrtus communis* L. by-product extracts in a Parkinson’s disease model. Unlike previous research focusing primarily on the antioxidant or anti-inflammatory properties of fresh myrtle extracts, this work explores the innovative use of waste from myrtle liqueur production as a sustainable source of bioactive molecules. These extracts exhibit a unique “cocktail effect”, where the synergy among bioactive compounds enhances their efficacy against oxidative damage and neuroinflammation, two central mechanisms in PD pathology [15,16]. Using by-products of *Myrtus communis* L. aligns with global efforts to foster a circular economy and mitigate environmental impact. Recent studies further suggest that polyphenols may critically regulate oxidative stress, mitochondrial dysfunction, and inflammatory pathways fundamental to PD progression [26,58,59,60]. Based on these considerations, the present study aimed to investigate whether polyphenols extracted from *Myrtus communis* L. by-products confer neuroprotective effects against 6-hydroxydopamine (6-OHDA)-induced toxicity in PC12 cells. These cells, derived from a rat adrenal pheochromocytoma, are widely employed as an in vitro model of Parkinson’s disease because they exhibit a dopaminergic phenotype, synthesize and release dopamine, and are highly susceptible to oxidative stress, hallmarks that closely mirror pathogenesis of PD [61,62,63]. This approach not only addresses pivotal gaps in the literature but also highlights the sustainable potential of these by-products as valuable resources for nutraceuticals or functional foods with the capacity to counteract neurodegenerative processes.

## 2. Materials and Methods

### 2.1. Reagents and Solvents

Analytical Standards of quercetin-3-O-galactoside, quercetin-3-O-rhamnoside, cyanidin-3-O-glucoside, petunidin-3-O-glucoside, peonidin-3-O-glucoside, malvidin-3-O-glucoside, and ellagic acid were purchased from Extrasynthese (Lyon, France). Gallic acid, 2,2-Diphenyl-1-picrylhydrazyl (DPPH), 2,4,6-tris(2-pyridyl)-s-triazine (TPTZ), ferric chloride, 6-hydroxy-2,5,7,8-tetramethylchromane-2-carboxylic acid (Trolox), sodium carbonate, Folin-Ciocalteau reagent, ferrous sulfate, TRIzol reagent, growth medium RPMI 1640, fetal bovine serum (FBS), 3-(4,5-Dimethyl-2-thiazolyl)-2,5-diphenyl-2H-tetrazolium Bromide (MTT), 2’,7’ -dichlorodihydrofluorescein diacetate (DCFH-DA), Nerve Growth Factor-β (NGF) from rat, dimethyl sulfoxide (DMSO), Poly-D-Lysine, and Penicillin-Streptomycin-Amphotericin B Suspension (100×) were purchased from Sigma-Aldrich (St. Louis, MO, USA). iScriptTM cDNA synthesis kit was purchased from Bio-Rad (Hercules, CA, USA). Gabrd (product number 249900, NM_008072, final conc. 1×), Grin1 (product number 249900, NM_017010, final conc. 1×), AChE (product number 249900, NM_172009, final conc. 1×), c-fos (product number 249900, NM_022197, final conc. 1×), PON2 (product number 249900, NM_001013082, final conc. 1×), and beta-actin (product number 249900, NM_007393, final conc. 1×) were purchased from Qiagen (Hilden, Germany). Ethanol absolute, methanol (LC-MS grade), acetonitrile for HPLC and orthophosphoric acid (ACS ISO, for analysis, 85%), and acetic acid glacial and sodium acetate anhydrous (ACS, Reag. Ph Eur) were purchased from Carlo Erba (Milan, Italy). Ultrapure water (conductivity lower than 18.2 MΩ) was distilled and filtered through a Milli-Q apparatus (Millipore, Milan, Italy).

### 2.2. Plant Materials

Exhausted myrtle barriers were kindly offered by a local company. In order to preserve thermolabile compounds, the berries were immediately brought to the laboratory, frozen and subsequently lyophilized by a LIO 5P DGT (Cinquepascal, Trezzano s/Naviglio (MI)).

### 2.3. Polyphenols Extraction

The extract was prepared using lyophilized material. The finely ground using a laboratory-grade grinder was thoroughly homogenized and divided into three replicates. For each replicate, 0.5 g of the powdered sample was mixed with 10 mL of 70% ethanol in 15 mL screw-capped Falcon tubes for the extraction of the phenolic fraction. Extraction was performed in an ultrasonic bath (ultrasonic cleaner 040S, AC220–240V 50 Hz, heating power 200 W, frequency 40 KHz, timer 0–30 min) for 60 min below 20 °C to prevent the degradation of polyphenols. The samples were then centrifuged at 4000 rpm for 10 min at 20 °C (Eppendorf Centrifuge 5810/5810 R). The resulting supernatant was diluted with a 0.22 M aqueous phosphoric acid solution and prepared for injection into the chromatographic system.

### 2.4. Polyphenols Analysis

An Agilent HPLC 1100 liquid chromatograph coupled with a Thermo Finnigan DAD CHROMQUEST UV 6000 diode array detector was used to perform the analysis. Chromatographic separation was obtained according to D’Amelia et al. [64]. The column was a Kinetex 5 µm (C18 100A—150 × 4.6 mm, Phenomenex). 10 µL volume sample was injected and eluted in 120 min (0.4 mL/min) via a binary gradient mobile phase consisting of H_3_PO_4_ 0.22 M (solvent A) and acetonitrile (solvent B) with a follow gradient elution: 0 min, 95% A–5% B; 30 min 90% A–10% B; 35 min, 85% A–15% B; 70 min, 70% A–30% B; 100 min, 10% A–90% B; 120 min, 100% B. Each time, the column was reconditioned for 15 min. Considering the chemical structure of main phenolic compounds, three distinct wavelengths were used: 280 nm, 360 nm and 520 nm. Peak identification was carried out by comparing each compound’s retention time and UV spectra with the reference standard. Individual phenolic compounds were quantified using an external standard calibration method. Hydrolysable tannins, without a specific standard, were expressed as gallic acid equivalents. Correlation values ranged between 0.9990 and 0.9999. A calibration curve was prepared in orthophosphoric acid 0.22 M from suitable dilution of standard stock solution (1000 mg/L in methanol).

### 2.5. Total Polyphenols

Total polyphenols determination was performed using the Folin-Ciocalteu method, following a modified protocol based on Singleton, V. L. (1965) [65]. 100 µL of the extract or standard (gallic acid) were added to 500 µL of Folin-Ciocalteau reagent and incubated for 5 min (room temperature); afterward, 3 mL of Na_2_CO_3_ 10% (p/v) and ultrapure water were added to reach a final volume of 10 mL. After 90 min of incubation at room temperature, the optical density (OD) was measured against a blank at 725 nm using a spectrophotometer, Varian Cary 50, and 1 cm wide disposable cuvettes. Quantitative analysis was carried out using an external standard calibration method, and the results were expressed in mg/kg of gallic acid equivalent (GAE).

### 2.6. Antioxidant Power Determinations

The DPPH (2,2-Diphenyl-1-picrylhydrazyl) assay was performed following a slightly modified protocol based on Brand-Williams et al. [66]. Then, 20 µL of the extract or the standard (Trolox) was added to 2 mL of a methanolic solution of DDPH at a concentration of 40 µM. After a 90 min incubation period at room temperature, the optical density (OD) was measured against a blank at 517 nm using 1 cm wide disposable cuvettes in a spectrophotometer Varian Cary 50. Quantitative analysis was carried out using an external standard calibration method, and the results were expressed in mM/kg of TEAC (Trolox equivalent antioxidant capacity).

The FRAP (Ferric Reducing Antioxidant Power) assay was performed following a slightly modified protocol based on Axelrod et al. [67]. The reagent was prepared by mixing TPTZ (10 mM) and ferric chloride (20 mM) in acetate buffer (pH 3.6). 50 μL of diluted extract 1:10 (*v*/*v*) or standard (ferrous sulfate) was added to 2 mL of this solution. After 4 min of incubation at room temperature, the OD was measured against a blank at 593 nm using 1 cm wide disposable cuvettes in a spectrophotometer Varian Cary 50. Quantitative analysis was carried out using an external standard calibration method, and the results were expressed in mmol/kg of ferrous sulfate.

### 2.7. Preparation of Myrtle Berry By-Product Extracts for Cell Assays

The lyophilized and finely ground material was extracted in 70% ethanol (*v*/*v*), maintaining the same matrix-to-solvent ratio as previously described in 2.3. The extracted sample was then centrifuged at 4000 rpm for 10 min at 20 °C (Eppendorf Centrifuge 5810/5810 R). The resulting supernatant was concentrated by rotary evaporation (Buchi R-300 Rotavapor System with V-700 Vacuum Pump and Chiller, BUCHI Italia s.r.l, Cornaredo, Italy) to remove residual ethanol and subsequently lyophilized by a LIO 5P DGT (Cinquepascal, Trezzano s/Naviglio (MI)) to obtain the final extract for use in cell-based assays.

### 2.8. Cell Cultures

PC12 (CRL-1721) cells were grown in RPMI 1640 medium supplemented with 2 mM glutamine, 10% horse serum, and 5% fetal bovine serum, including 1% penicillin and streptomycin solution, at 37 °C with 5% CO_2_. Cells were harvested when 80% confluence was reached. For each experiment PC12 cells were seeded in 96-well plates pre-coated with poly-D-lysine. The differentiation medium was also based on RPMI-1640 with 0.1% FBS and with the addition of NGF at concentrations of 100 ng/mL. The culture medium was changed every 48 h for 7 days [68]. All experiments were carried out between passages 10–20.

### 2.9. Experimental Design

In order to create a model of toxicity, increasing concentrations of 6OHDA (5, 10, 25, 50, 100 µM) were added, and the IC50 was determined. In order to evaluate the neuroprotective properties of the myrtle extract, cells were pre-treated with increasing concentrations of extract (10, 25, 50 and 100 µg/mL) for 12 h and then exposed to 6-hydroxydopamine IC50 for 24 h. Cells treated with the same volume of RPMI 1640 medium alone were used as a control.

### 2.10. MTT Assay

PC12 cells were plated into 96-well plates at 1 × 10^4^ cells per well and incubated as previously described in 2.7. paragraph to induce cell adhesion. Cell viability was assessed using MTT assay [69], and 20 μL of MTT (2.5 mg/mL) was added and incubated for 4 h in the dark. The supernatants were cautiously taken out, and 100 μL DMSO was added to each well to liquefy the formazan precipitate. The absorbance of each well at a wavelength of 570 nm was measured with a microplate reader (Victor X5 2030 Multilabel HTS Fluorescence, Perkin Elmer, Milano, Italy). Results were expressed as % of positive control.

### 2.11. ROS Determination

ROS formation was measured by using the cell-permeable indicator 2′,7′-dichlorodihydrofluorescein diacetate (DCFH-DA) [70]. The stock solution of DCFH-DA was prepared in DMSO at a concentration of 25 mM and stored at −20 °C. 1 × 10^4^ cells suspension was seeded into a 96-well microplate and incubated with 25 µM of DCFH-DA solution for 30 min at 37 °C in the dark. The fluorescence of 2′,7′-dichlorofluorescein (DCF) was measured with a microplate reader (Perkin Elmer Victor X5 2030 Multilabel HTS Fluorescence) at room temperature, with excitation and emission filters of 485 and 530 nm, respectively. The total DCF fluorescence data were corrected by subtracting the background fluorescence. Results were expressed as % of positive control.

### 2.12. Real-Time RT-PCR

PC12 cells were seeded at a density of 1 × 10^6^ cells/mL in a T75 flask for 24 h. At the end of treatment, the cells were harvested, and total RNA was extracted using the TRIzol reagent following the manufacturer’s instructions. RNA concentration was estimated at 260 nm using a Thermo Scientific NanoDrop spectrophotometers. From each sample, 1 µg of RNA was employed for single-strand cDNA synthesis using an iScriptTM cDNA synthesis kit according to the manufacturer’s instructions. cDNA was used as a template for RT-PCR to determine the expression of the following genes: Gabrd (GABAA receptor delta subunit), Grin1 (NMDA receptor NR1 subunit, AChE, c-fos, PON2, and beta-actin rRNA as a housekeeping gene. Amplification was performed with a Bio-Rad c1000 thermocycler with the following conditions: initial heating at 94 °C for 5 min to denature the cDNA and activate the Taq DNA Polymerase, followed by 95 °C for 30 s, 60 °C for 60 s, 72 °C for 45 s for 34 cycles followed by a final step at 72 °C for 5 min. The target Ct values were normalized to beta-actin, used as the reference gene. The mRNA levels in PC12 cells subjected to various treatments were calculated using the 2^(−ΔΔCt) method relative to the mRNA levels in untreated PC12 cells. These levels were expressed as a percentage relative to the control group.

### 2.13. Statistical Analysis

Each experiment was independently repeated at least three times, and the results are presented as mean ± SD. Statistical analysis was conducted using GraphPad PRISM 8.00 (GraphPad Software, San Diego, CA, USA). The means and standard deviations (SD) were derived from three separate experiments. Data analysis utilized ANOVA Dunnett’s test to compare multiple groups against the control group while adjusting for cumulative alpha error. Significance levels, indicated by asterisks (*), are based on *p*-values. No asterisk denotes a *p*-value > 0.05; * indicates *p*-value < 0.05; ** *p*-value < 0.01; *** *p*-value < 0.001; and **** *p*-value < 0.0001.

## 3. Results and Discussion

### 3.1. Total Polyphenols, Antioxidant Activity, and HPLC Characterization

A preliminary test of polyphenol total content was carried out to evaluate the presence of phenolic compounds in the exhausted berries. The total polyphenol (TP) content, reported in Table 1, was expressed as g GAE/kg of dry extract. With the high TP amount of 42.91 ± 1.32 g/kg, the analyzed samples showed quantities of polyphenols comparable to those detected in fresh berry samples, as reported by Wannes and Marzouk [71], confirming that they can be a candidate as secondary material for the extraction of bioactive compounds. We then proceeded with an antioxidant activity evaluation by the DPPH radical scavenging and Ferric Reducing Antioxidant Power assays, whose results are reported in Table 1. This high TP content likely contributes to the extract’s notable antioxidant activity, as indicated by a Trolox equivalent antioxidant capacity (TEAC) of 270.45 ± 0.26 mmol/kg and a Ferric Reducing Antioxidant Power (FRAP) of 953.29 ± 20.66 mmol/kg. As the antioxidant activity is positively correlated with the phenolic amount of extract, it was easy to hypothesize that it is greatly influenced by the phenolic composition of the sample.

**Figure 1:**
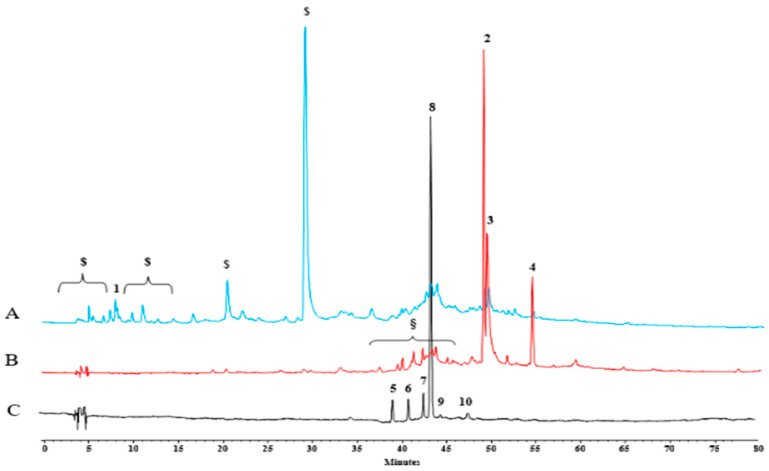
HPLC-DAD chromatogram 280 nm (A), 360 nm (B) and 520 nm (C) exhausted myrtle berries. (1) Gallic acid, (2) Hyperoside, (3) Ellagic acid, (4) Quercitrin, (5) Cyanidin 3-O-glucoside, (6) Petunidin 3-O-glucoside, (7) Peonidin 3-O-glucoside, (8) Malvidin 3-O-glucoside, (9) Petunidin 3-O-arabinoside, (10) Malvidin 3-O-arabinoside; ($) Gallotannins; (§) Ellagitannins.

For this reason, an HPLC analysis of the hydroalcoholic extract was performed with the aim of identifying the main polyphenols. Chromatograms reported in Figure 1 show that the extract was rich in hydrolyzable tannins, ellagic and gallic acid. Peaks in the chromatogram acquired at 360 nm and 520 nm corresponded with the quercetin and anthocyanin derivatives, respectively.

As reported in Table 2, the quantitative analysis of the primary phenolic compounds identified in myrtle berries by-products reveals that the phenolic profile was dominated by hydrolyzable tannins, which comprise over 90% of the total phenolic content (6051.18 ± 370.52 mg/kg_DW_). Phenolic acids were identified in lower concentrations and were characterized mainly by gallic acid (127.25 ± 0.34 mg/kg_DW_) and ellagic acid at a relatively high concentration (546.12 ± 42.37 mg/kg_DW_). A small amount of flavonoid was quantified, represented by quercetin-3-O-galactoside and quercetin-3-O-rhamnoside at the concentration of 40.80 ± 1.03 mg/kg_DW_ and 25.33 ± 1.40 mg/kg_DW_, respectively.

The extract analyzed showed, even if in a lower concentration (<1%), also anthocyanins compounds where malvidin-3-O-glucoside was the most important with 25.68 ± 2.34 mg/kg_DW_. Thus, the extract’s antioxidant capacity is reasonably due to the high levels of hydrolyzable tannins, gallic acid, and ellagic acid, identified by chromatographic analysis. Tannins compounds are well-established for their antioxidant and metal-chelating properties, which are pivotal in mitigating oxidative stress, a critical factor in the pathogenesis of neurodegenerative diseases such as Parkinson’s disease (PD) [60,72]. Extensive research has demonstrated the neuroprotective effects of hydrolyzable tannins, particularly through the inhibition of reactive oxygen species (ROS) and the attenuation of neuroinflammation, key mechanisms in the progression of PD [73,74]. By modulating inflammatory responses, including the suppression of TNF-α and IL-6, tannins play an essential role in preserving neuronal survival [75]. Given their concentration in the myrtle extract, it is likely that hydrolyzable tannins contribute substantially to the observed protection against 6-OHDA-induced cytotoxicity in PC12 cells. The neuroprotective potential of gallic and ellagic is well-documented. In PD, gallic acid’s potent antioxidant capacity plays a crucial role in counteracting oxidative stress, a key contributor to dopaminergic neuron degeneration. In addition to reducing oxidative damage, gallic acid exerts anti-inflammatory effects, further reinforcing its neuroprotective efficacy [76,77,78]. This dual action mitigating both oxidative and inflammatory damage positions gallic acid as a vital component of the extract’s overall efficacy.

The presence of ellagic acid further amplifies the extract’s antioxidant profile. Ellagic acid is well-known for its ability to scavenge free radicals and inhibit lipid peroxidation, with neuroprotective effects demonstrated in various models of neurodegeneration. In the MPTP (1-metil 4-fenil 1,2,3,6-tetraidropiridina) model of PD, ellagic acid has been shown to prevent dopaminergic neuron degeneration by lowering oxidative stress markers such as malondialdehyde (MDA) and restoring antioxidant enzyme levels, including superoxide dismutase (SOD) and glutathione (GSH) [79].

Similarly, flavonoid glycosides exhibit potent antioxidant properties. These compounds modulate oxidative stress through the activation of the Nrf2 pathway and inhibition of NF-κB-mediated inflammation, mechanisms crucial for neuroprotection. Moreover, their inhibition of pro-inflammatory enzymes such as COX and LOX enhances their anti-inflammatory effects, providing additional neuroprotective benefits in diseases like PD [80,81]. Anthocyanins demonstrate significant antioxidant activity neutralizing ROS and mitigating oxidative stress by upregulating endogenous antioxidant defenses, such as SOD and glutathione peroxidase (GSH-Px) while inhibiting lipid peroxidation [82]. Moreover, anthocyanins exert significant neuroprotective effects by modulating critical molecular pathways, particularly Nrf2 (nuclear factor erythroid 2-related factor 2) and NF-κB (nuclear factor kappa B). Research indicates that dietary intake of anthocyanins enhances Nrf2 expression in the hippocampus and prefrontal cortex of aging rats, which is associated with increased activity of antioxidant enzymes. Additionally, in vitro studies have shown that certain anthocyanins, such as cyanidin-3-glucoside, promote Nrf2 activation, leading to a reduction in oxidative stress and the prevention of neuronal apoptosis. These compounds also strongly inhibit the NF-κB pathway by stabilizing its endogenous inhibitor, IκB-α, thereby blocking its degradation and subsequent translocation of NF-κB to the nucleus. Experimental findings highlight that anthocyanins suppress the release of pro-inflammatory mediators, including nitric oxide (NO) and prostaglandin E2 (PGE2), in microglial cells stimulated by lipopolysaccharides (LPS). Furthermore, anthocyanins lower the production of pro-inflammatory cytokines such as TNF-α and IL-1β and mitigate the activation of upstream regulators of NF-κB, such as the AKT/JNK pathway [83,84].

### 3.2. Effect of the Extract on Cell Viability and ROS Levels

**Figure 2:**
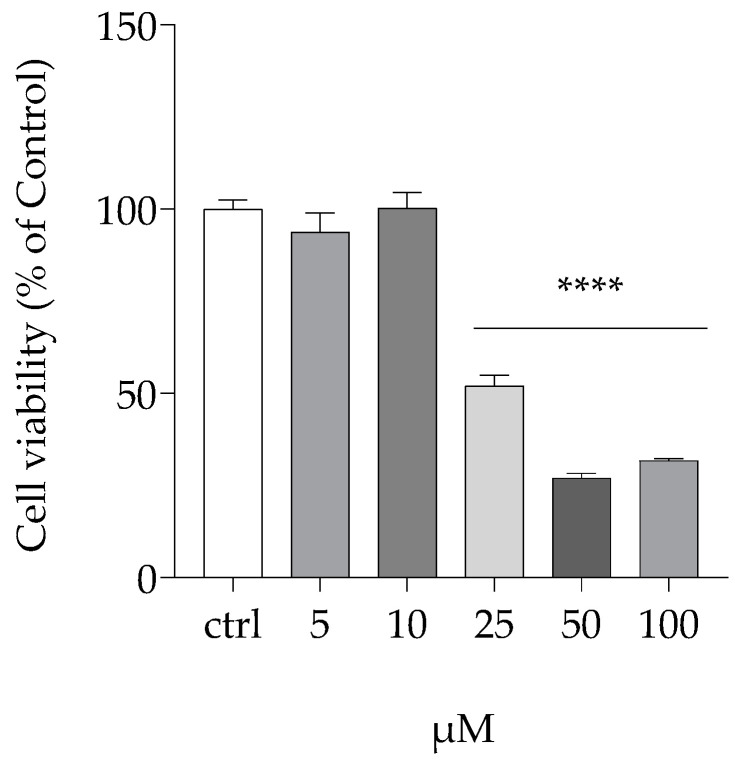
Cytotoxic effect of 6-hydroxydopamine on PC12 cell viability. Mean differences were compared using one-way ANOVA with Dunnet’s multiple comparisons test (n = 12, **** *p* < 0.0001).

The impact of the myrtle berry extract on PC12 cell viability and intracellular ROS levels provides important insights into its potential as a cytoprotective and antioxidant agent, particularly in PD cellular models. Figure 2 demonstrates the cytotoxic effects of 6-hydroxydopamine (6OHDA) on PC12 cells, a well-established model for studying PD-related neurotoxicity [85]. After exposure to varying concentrations of 6OHDA (5, 10, 25, 50, and 100 µM) for 24 h, a significant reduction in cell viability was observed, particularly at 25 µM, where a 50% decrease was noted.

**Figure 3:**
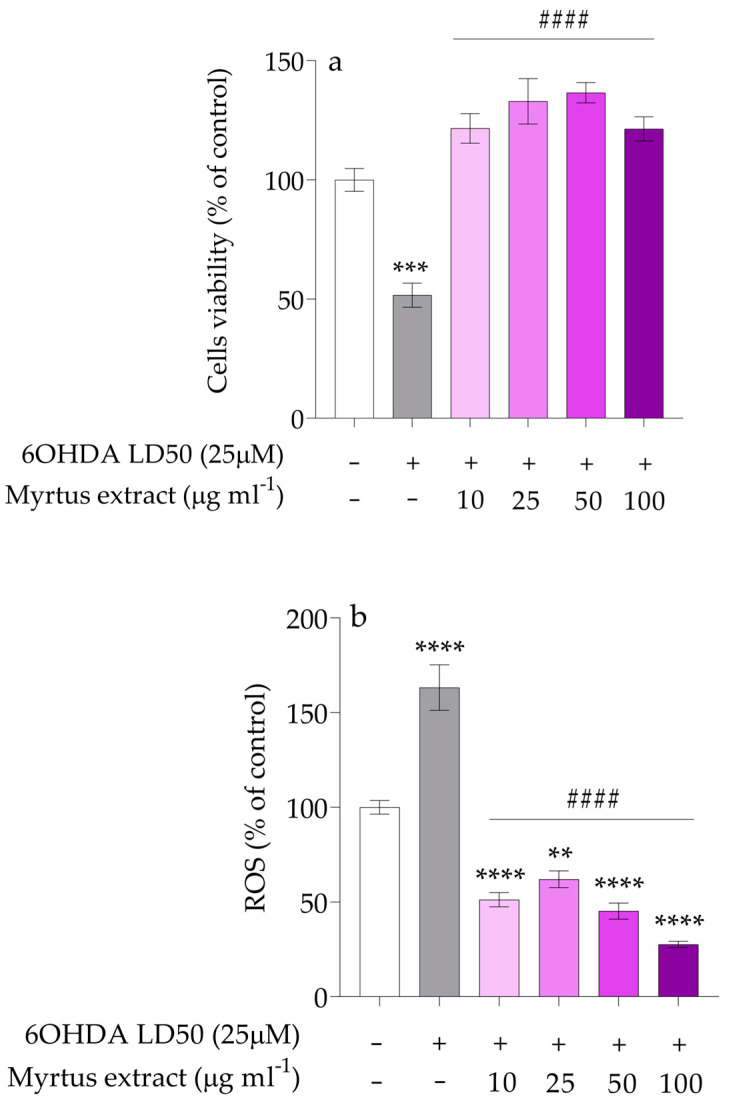
(**a**) Protective effect of extract against 6OHDA on PC12 cell viability. The white bars represent the control group, while the gray bars represent the group treated with 6OHDA at the lethal dose 50 (25 μM). The remaining bars correspond to groups pre-treated with Myrtus extract at the indicated concentrations (10, 25, 50, and 100 μg mL^−1^) for 12 h, followed by treatment with 6OHDA for 24 h. Mean differences were compared using one-way ANOVA with Dunnet’s multiple comparisons test (n = 12, *** *p* < 0.001; #### *p* < 0.0001); (**b**) ROS levels after oxidative stress induced by 6OHDA. Mean differences were compared using one-way ANOVA with Dunnet’s multiple comparisons test (n = 12, ** *p* < 0.01; **** *p* < 0.0001; #### *p* < 0.0001).

Given the pivotal role of oxidative stress in the pathogenesis of Parkinson’s disease (PD), administering the extract may be recognized as an effective strategy for mitigating mitochondrial oxidative damage. As illustrated in Figure 3a, pre-treatment with different concentrations of myrtle extract (10, 25, 50, and 100 µg/mL) for 12 h, even at the lowest concentrations, significantly protects against cell death exposed to 6OHDA for 24 h. At the highest concentrations, the extract not only shields cytotoxic effects but also augments cell survival over baseline levels. Comparable results have been reported in studies using other natural antioxidant extracts, which also enhance cell survival by mitigating oxidative damage and activating stress-response pathways. However, the ability of myrtle extract to augment cell survival above baseline levels highlights its unique therapeutic potential [86,87].

The 6OHDA treatment led to a significant increase in intracellular ROS levels (**** *p* < 0.0001), as shown in Figure 3b. Interestingly, treatment with the myrtle extract not only significantly reduced ROS levels but also lowered them to values below those observed in the control group (#### *p* < 0.0001), highlighting its strong antioxidant properties in combating oxidative stress. This result is comparable to findings from other studies conducted on different cell lines, further underscoring the potent antioxidant properties of the extract in combating oxidative stress and highlighting its potential as a neuroprotective agent [88]. Its ability to nearly restore ROS levels to normal emphasizes its protective role against oxidative damage, reinforcing its potential as a neuroprotective agent. These findings suggest that myrtle berry extract has promising cytoprotective effects in addressing PD-related oxidative stress by improving cell viability and reducing ROS levels. This aligns with existing research, which highlights the neuroprotective benefits of polyphenols, especially in neurodegenerative diseases like Parkinson’s.

### 3.3. Oxidative Stress-Related Gene Modulation

Growing evidence suggests that the biological effects of polyphenols extend beyond their well-established antioxidant activity; in fact, they also modulate metabolic pathways, cellular signaling, and gene expression. Numerous genes are crucial in managing oxidative stress and providing neuroprotection, especially in the context of neurodegenerative diseases where oxidative damage is a major contributing factor. Among these, AChE, PON2, NMDA (N-methyl-D-aspartate) receptor NR1 subunit, GABA (gamma-aminobutyric acid) type A receptor delta subunit, and c-fos play distinct yet interconnected roles in maintaining neuronal homeostasis and modulating synaptic function. Together, their actions support cellular resilience under stress, ensuring the balance of excitatory and inhibitory signaling essential for brain health.

**Figure 4:**
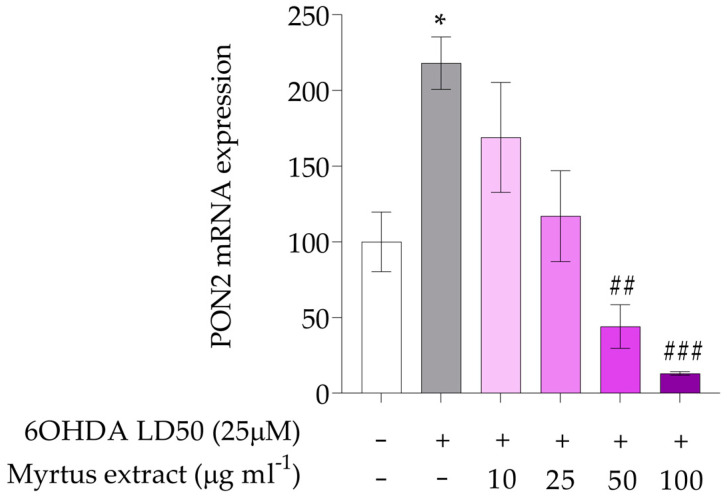
Gene expression of PON2. The white bars represent the control group, while the gray bars represent the group treated with 6OHDA at the lethal dose of 50 (25 μM). The remaining bars correspond to groups pre-treated with Myrtus extract at the indicated concentrations (10, 25, 50, and 100 μg mL^−1^) for 12 h, followed by treatment with 6OHDA for 24 h. The mRNA levels for PON2 gene were expressed as % of control observed in untreated PC12 (mean ± SD; n = 3) and normalized to beta-actin. Mean differences were compared using one-way ANOVA with Dunnet’s multiple comparisons test (* *p* < 0.05; ## *p* < 0.01; ### *p* < 0.001).

In our experiments, PON2 expression (Figure 4) was affected by oxidative stress in cells treated with 6-OHDA, a neurotoxin commonly used to model Parkinson’s disease pathophysiology. Oxidative stress caused a significant upregulation of PON2 (* *p* < 0.05), consistent with its role as a stress-response enzyme. However, pre-treatment with myrtle berry extracts 50 and 100 μg mL^−1^ significantly (## *p* < 0.01; ### *p* < 0.001) mitigated this upregulation, suggesting that the neuroprotective properties of these extracts may partially rely on their ability to regulate PON2 expression. By preventing excessive PON2 activation, myrtle berry extracts could help maintain redox homeostasis, reduce oxidative damage, and promote overall cellular health.

PON2 is differentially expressed in the brain, with particularly high levels in dopaminergic regions such as the striatum and substantia nigra, which are critically affected in Parkinson’s disease. Previous studies have shown that PON2 expression increases in response to oxidative stress and correlates with elevated levels of reactive oxygen species (ROS) [89,90]. This indicates that PON2 plays a key role in maintaining cellular homeostasis by acting as an antioxidant and protecting cells from oxidative damage [91]. Its intracellular localization, particularly in mitochondria and the endoplasmic reticulum (ER), allows PON2 to lower ROS levels and prevent apoptosis triggered by oxidative stress. Additionally, PON2 alleviates ER stress by reducing the accumulation of misfolded proteins and suppressing pro-apoptotic pathways, a mechanism particularly relevant in neurodegenerative diseases such as Parkinson’s disease [92]. PON2’s protective role in dopaminergic neurons is further highlighted by its ability to counteract oxidative damage caused by neurotoxins like 6-OHDA and MPTP, which induce mitochondrial dysfunction and ROS accumulation. Enhancing PON2 activity has been shown to improve neuronal survival in these models, underscoring its therapeutic potential [93]. Polyphenols, known for their strong antioxidant properties, have been shown to modulate PON2 expression and activity. For instance, quercetin, a well-studied polyphenol, has been shown to regulate PON2 activity, enhancing cellular defenses against oxidative stress. This modulation is particularly important in the context of neurodegenerative diseases, where oxidative stress and mitochondrial dysfunction are key drivers of disease progression [92].

Beyond its antioxidant activity, PON2 can also mitigate ER stress and prevent neuronal apoptosis by regulating key stress-response proteins such as CHOP and GRP78. Furthermore, transcription factors like FOXA1 have been identified as regulators of PON2 expression, further enhancing its protective effects in neurodegenerative conditions. By upregulating PON2, FOXA1 reduces oxidative and ER stress, ultimately mitigating dopaminergic neuronal loss [92]. These findings emphasize the importance of maintaining optimal PON2 expression for neuronal health and highlight its potential as a therapeutic target.

Our findings contribute to this growing body of evidence, suggesting that the neuroprotective effects of myrtle berry extracts may be mediated, at least in part, through their ability to modulate PON2 activity and expression. By reducing oxidative stress, preserving mitochondrial function, and alleviating ER stress, these extracts could play a significant role in protecting dopaminergic neurons and slowing the progression of neurodegenerative diseases such as Parkinson’s disease.

**Figure 5:**
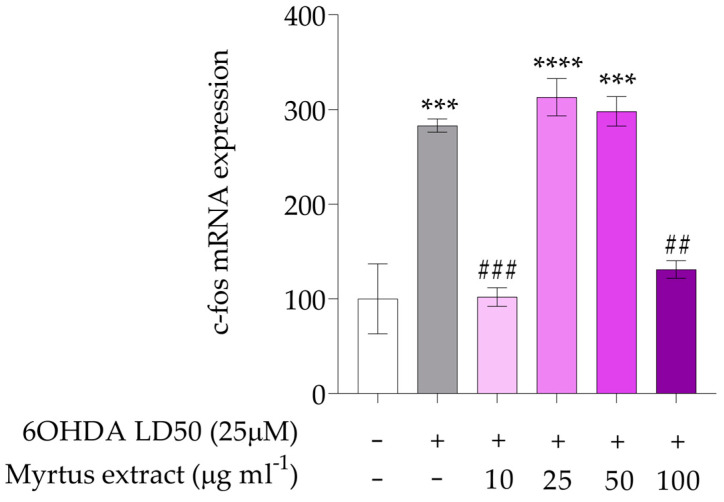
Gene expression of c-fos. The white bars represent the control group, while the gray bars represent the group treated with 6OHDA at the lethal dose of 50 (25 μM). The remaining bars correspond to groups pre-treated with Myrtus extract at the indicated concentrations (10, 25, 50, and 100 μg mL^−1^) for 12 h, followed by treatment with 6OHDA for 24 h. The mRNA levels for c-fos were expressed as % of control observed in untreated PC12 (mean ± SD; n = 3) and normalized to beta-actin. Mean differences were compared using one-way ANOVA with Dunnet’s multiple comparisons test (*** *p* < 0.001; **** *p* < 0.0001; ## < 0.01; ### *p* < 0.001).

The expression of c-fos, an immediate early gene commonly used as a marker for neuronal activity, shows, as reported in Figure 5, notable increases in both treatments with 6OHDA (*** *p* < 0.001) and the extracts derived from myrtle at the dose of 25 and 50 μg mL^−1^ (*** *p* < 0.001; **** *p* < 0.0001). This elevation in c-fos levels suggests that 6OHDA, a known neurotoxin, triggers significant neuronal activity or stress responses, indicating the cellular mechanisms that neurons employ to cope with neurotoxic effects. This response may reflect alterations in signaling pathways that contribute to neurodegeneration. In contrast, the increase in c-fos expression with the myrtle extracts may imply that these extracts exert a neuroprotective effect or promote adaptive responses in neuronal cells. The activation of c-fos by the extracts could indicate an enhancement of neuronal resilience or the activation of protective signaling pathways. Understanding how these extracts modulate c-fos expression in the context of neurotoxicity may provide valuable insights into their mechanisms of action. c-fos also plays a crucial role in the nervous system, essential for neuronal excitability and survival. c-fos is a protein that functions as a transcription factor, playing a crucial role in regulating gene expression. It is part of the AP-1 (Activator Protein-1) complex, which is involved in various cellular processes, including proliferation, differentiation, and response to stress. Its expression can be rapidly induced in response to external stimuli, and it often acts in conjunction with other transcription factors to modulate gene expression. Research has indicated that polyphenols may influence the expression of various genes, including those regulated by transcription factors like c-fos. For instance, polyphenols can modulate the activity of c-fos and the AP-1 complex, potentially affecting cellular responses to oxidative stress and inflammation [94,95].

**Figure 6:**
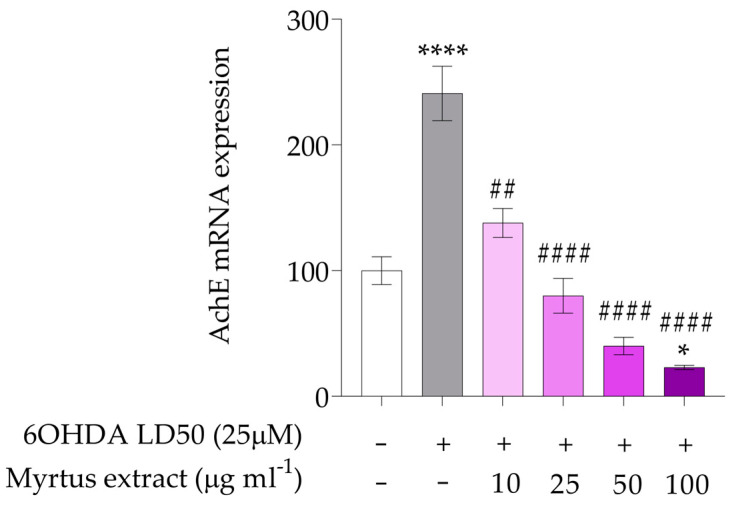
Gene expression of AChE. The white bars represent the control group, while the gray bars represent the group treated with 6OHDA at the lethal dose of 50 (25 μM). The remaining bars correspond to groups pre-treated with Myrtus extract at the indicated concentrations (10, 25, 50, and 100 μg mL^−1^) for 12 h, followed by treatment with 6OHDA for 24 h. The mRNA levels for AChE gene were expressed as % of control observed in untreated PC12 (mean ± SD; n = 3) and normalized to beta-actin. Mean differences were compared using one-way ANOVA with Dunnet’s multiple comparisons test (* *p* < 0.05; **** *p* < 0.0001; ## < 0.01; #### *p* < 0.0001).

In our study, as shown in Figure 6, the expression of AChE was evaluated in cells pre-treated with the extracts (10, 25, 50 and 100 µg/mL) for 12 h and then exposed to 6OHDA. 6OHDA up-regulated mRNA levels (**** *p* < 0.0001) contrarily to extract pre-treated cells where the mRNA levels were significantly downregulated, indicating a biphasic effect of the extract. Decreased levels of acetylcholinesterase gene expression lead to increased levels of acetylcholine, which can enhance cholinergic neurotransmission. Polyphenols, such as curcumin and polyphenols in orange peel, have demonstrated acetylcholinesterase inhibitory activity and gene expression downregulation [96,97]. Acetylcholine is a key neurotransmitter involved in many physiological functions, including learning, memory, and muscle activation. 6-OHDA induces apoptosis [98,99], a process that is also influenced by the integrity of acetylcholinesterase (AChE), which plays a crucial role in terminating signal transmission in the cholinergic system and is implicated in the pathogenesis of neurodegenerative diseases through its effects on inflammation, oxidative stress, and protein aggregation [100]. AChE genes have been implicated in aging processes and other neurodegenerative diseases. Studies have shown that reduced AChE expression decreases apoptotic markers in various cell lines, whereas increased AChE activity promotes apoptosis [101,102].

**Figure 7:**
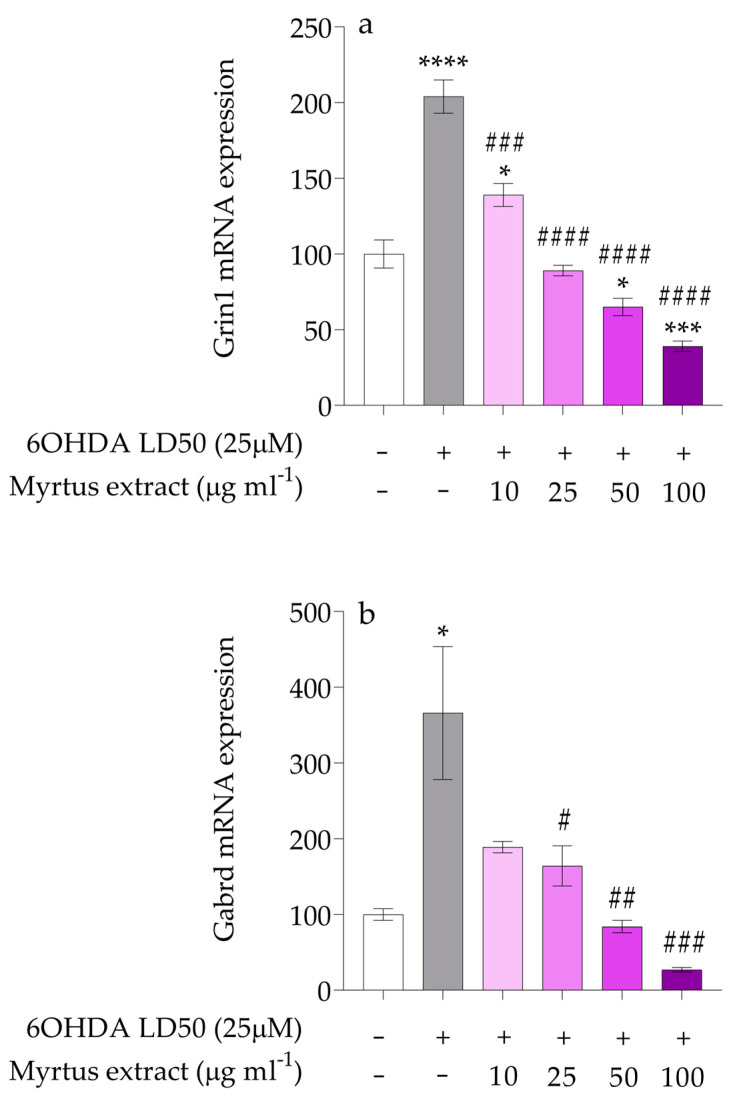
(**a**) Gene expression of Grin1. The white bars represent the control group, while the gray bars represent the group treated with 6OHDA at the lethal dose of 50 (25 μM). The remaining bars correspond to groups pre-treated with Myrtus extract at the indicated concentrations (10, 25, 50, and 100 μg mL^−1^) for 12 h, followed by treatment with 6OHDA for 24 h. The mRNA levels for Grin1 gene were expressed as % of control observed in untreated PC12 (mean ± SD; n = 3) and normalized to beta-actin. Mean differences were compared using one-way ANOVA with Dunnet’s multiple comparisons test (* *p* < 0.05; *** *p* < 0.001; **** *p* < 0.000; ### *p* < 0.001; #### *p* < 0.0001); (**b**) Gene expression of Gabrd. The white bars represent the control group, while the gray bars represent the group treated with 6OHDA at the lethal dose of 50 (25 μM). The remaining bars correspond to groups pre-treated with Myrtus extract at the indicated concentrations (10, 25, 50, and 100 μg mL^−1^) for 12 h, followed by treatment with 6OHDA for 24 h. The mRNA levels for the Gabrd gene were expressed as % of control observed in untreated PC12 (mean ± SD; n = 3) and normalized to beta-actin. Mean differences were compared using one-way ANOVA with Dunnet’s multiple comparisons test (* *p* < 0.05; # *p* < 0.05; ## *p* < 0.01; ### *p* < 0.001).

The expression of Grin1 coding for NMDA receptor NR1 subunit was assessed in PC12 cells pre-treated with different concentrations of waste extracts for 12 h before exposure to 6OHDA. Results reported in Figure 7a showed significant downregulation of Grin1 mRNA levels in cells pre-treated with the extracts, compared with those treated with 6OHDA alone, which in contrast shows upregulation (**** *p* < 0.0001) as also demonstrated by other studies. Polyphenols have also been studied for their effects on the NMDA receptor, particularly the NR1 subunit. The NR1 subunit is essential for the proper functioning of the NMDA receptor, as it is required for channel formation and is present in all functional NMDA receptors. Through their antioxidant and anti-inflammatory properties, polyphenols may regulate the activity of NMDA receptors, reducing excitotoxic stress on neurons. Certain polyphenols have been found to provide neuroprotective benefits through the modulation of glutamate receptor activity and expression. For example, resveratrol has been shown to alleviate glutamate-induced excitotoxicity. When applied at concentrations ranging from 10 to 100 μM in hippocampal CA1 neurons, resveratrol significantly inhibited both the amplitude and frequency of postsynaptic currents mediated by glutamate receptors [103]. In another study utilizing selective inhibitors for glutamate receptors in hippocampal slices, resveratrol demonstrated potent antioxidant and scavenging properties [104]. The NMDA receptor is a type of glutamate receptor, which is the primary excitatory neurotransmitter in the central nervous system. This receptor plays a crucial role in synaptic plasticity, learning, and memory. Overexpression of the NMDA receptor is also associated with neuronal inflammation and increased oxidative stress, both of which contribute to the progression of neurodegenerative diseases. Some polyphenols can modulate NMDA receptor activity, reducing hyperactivation and thus limiting the risk of excitotoxicity [105]. Additionally, maintaining balanced neurotransmission is critical for the nervous system to function properly. Even slight, prolonged disruptions can trigger negative feedback mechanisms that may contribute to the development of various neurological disorders. Among the many potentially harmful factors, oxidative stress and altered metabolism of key neurotransmitters like GABA and glutamate are particularly impactful [106]. For these reasons, in the next experiments, after evaluating the expression of the NR1 subunit of the NMDA receptor, we evaluated the expression of the delta subunit of the GABAA receptor.

The results obtained (Figure 7b) indicate that pre-treatment with myrtle extracts significantly affects the expression of the delta subunit of the GABAA receptor in PC12 cells subsequently exposed to 6OHDA (* *p* < 0.05). Notably, this downregulation appears to be dose-dependent, as higher concentrations of myrtle berry extracts (50 and 100 µg/mL) resulted in more pronounced reductions in delta subunit expression compared with lower concentrations (10 and 25 µg/mL). This finding underscores the potential therapeutic implications of myrtle extracts, indicating that optimizing the dosage could enhance their neuroprotective effects against 6OHDA-induced toxicity. The upregulation of the delta subunit of the GABAA receptor may serve as a compensatory response to the overexpression of the NR1 subunit of the NMDA receptor induced by 6-OHDA treatment. This heightened NMDA activity can lead to excitotoxic stress, prompting a compensatory increase in the GABAergic system to balance neuronal excitability. The delta subunit of the GABAA receptor regulates tonic inhibition, providing a persistent inhibitory influence that can counterbalance the effects of NMDA receptor overactivity. Therefore, the observed increase in the delta subunit likely represents a neuroprotective adaptation aiming to stabilize excitatory-inhibitory signaling and prevent excitotoxic damage in response to stress. Polyphenols have been shown to modulate the function of GABAA receptors, including those containing the delta subunit. This modulation could potentially lead to calming effects, reduced anxiety, and improved sleep, as polyphenols like flavonoids have been shown to interact with GABAergic systems. For example, flavonoids found in foods like tea, fruits, and wine have been investigated for their ability to modulate GABAA receptor activity. Some polyphenols may act similarly to neurosteroids, which enhance GABAergic signaling by binding to specific receptor subunits, including delta. By modulating the delta subunit-containing receptors, polyphenols might contribute to the maintenance of a healthy balance between excitation and inhibition in the brain [107]. This balance is particularly critical in the context of neurodegenerative disorders like Parkinson’s disease, where disruptions in GABAergic signaling and excitatory-inhibitory homeostasis exacerbate neuronal vulnerability. By modulating these pathways, polyphenols present a promising therapeutic strategy to stabilize neuronal activity and reduce excitotoxicity.

Further research is needed to fully elucidate the mechanisms by which polyphenols influence neuronal receptors and their clinical implications. This study highlights the significant potential of *Myrtus communis* L. extracts to mitigate oxidative stress and neuroinflammation, two key drivers of Parkinson’s disease pathology. However, this work has certain limitations that should be addressed in future studies. A primary limitation is the lack of validation in in vivo models, which are essential to confirm the extract’s efficacy within complex biological systems. While in vitro models like the PC12 cell line provide controlled environments to study molecular mechanisms, they cannot fully replicate the complexities of the human nervous system. Future research should focus on animal models to assess the extract’s bioavailability, its ability to cross the blood–brain barrier (BBB), and its effects on behavioral and motor symptoms characteristic of Parkinson’s disease. Translating these findings into clinical applications will also require addressing several critical factors. The bioavailability of phenolic compounds and their metabolites plays a pivotal role in determining in vivo efficacy. For example, hydrolyzable tannins, such as those identified in this extract, are metabolized into bioactive molecules like ellagic acid derivatives, which can cross the BBB and exert neuroprotective effects. Advanced delivery systems, such as nanoformulations, should be explored to enhance the bioavailability of these compounds and ensure effective BBB penetration. The neuroprotective potential of *Myrtus communis* L. extracts may further benefit from the synergistic action of their diverse phenolic compounds. Hydrolysable tannins, flavonoids, and anthocyanins work together to amplify antioxidant and anti-inflammatory pathways, offering a multi-targeted approach to combating oxidative stress and neuroinflammation. By addressing these aspects, *Myrtus communis* L. by-product extracts could serve as a sustainable and effective resource for nutraceutical interventions in neurodegenerative diseases.

## 4. Conclusions

Our findings demonstrate the neuroprotective effects of myrtle berry waste extracts on PC12 cells under 6OHDA-induced stress, particularly through the modulation of specific genes. The complex profile of phenolic compounds in the myrtle extract, including hydrolyzable tannins, phenolic acids, flavonoid glycosides, and anthocyanins, highlights a synergistic effect that provides strong antioxidant and anti-inflammatory properties. Together, these compounds may effectively protect neuronal cells from oxidative stress and inflammation, two key drivers of neurodegenerative disease processes. To our knowledge, this is the first study to evaluate the neuroprotective effects of *Myrtus communis* L. berry by-products in a Parkinson’s disease model. Unlike previous research that focused primarily on the antioxidant or anti-inflammatory properties of fresh myrtle extracts, this work emphasizes the potential of valorizing food waste from myrtle liqueur production. The findings highlight the capacity of these extracts to mitigate oxidative stress, modulate key genes involved in neuroinflammation, and contribute to neuronal resilience, offering a novel perspective on the therapeutic applications of myrtle-derived polyphenols. Future studies should focus on validating these findings in in vivo models, exploring the pharmacokinetics and bioavailability of bioactive compounds, and developing advanced formulations to optimize their delivery to the central nervous system. By addressing these aspects, *Myrtus communis* L. berry by-products could serve as a sustainable and effective resource for nutraceutical and therapeutic interventions in neurodegenerative diseases, aligning with global efforts to promote a circular economy and environmental sustainability.

## Figures and Tables

**Table 1:** Total phenolic compounds and antioxidant power quantified in exhausted myrtle berries. All average contents with their standard deviations (n = 3 replicates) were expressed as dry weight.

TP	DPPH	FRAP
g/kg	mmol/kg	mmol/kg
42.91 ± 1.32	270.45 ± 0.26	953.29 ± 20.66

**Table 2:** Phenolic compounds quantified in exhausted myrtle berries. Average content (mg/kgDW) with their standard deviations, n = 3 replicates.

RT (min)	Compound	Concentration mg/kg
	**280 nm**	
8.60	Gallic acid	127.25 ± 0.34
-	Hydrolysable tannins *	6051.18 ± 370.52
48.8	Ellagic acid	546.12 ± 42.37
	**360 nm**	
48.2	Quercetin-3-O-galactoside	40.80 ± 1.03
53.2	Quercetin-3-O-rhamnoside	25.33 ± 1.40
	**520 nm**	
39.5	Cyanidin-3-galactoside	1.36 ± 0.00
41.5	Cyanidin-3-glucoside	0.71 ± 0.16
43.3	Petunidin-3-glucoside	7.23 ± 0.12
44.9	Peonidin-3-glucoside	2.97 ± 0.23
48.5	Malvidin-3-glucoside	25.68 ± 2.34

* Hydrolysable tannins concentrations were expressed as gallic acid equivalent.

## Data Availability

Data will be available upon request.
